# Regenerative medicine: potential of stem cells and tissue engineering in organ reconstruction

**DOI:** 10.1097/MS9.0000000000004367

**Published:** 2025-11-22

**Authors:** Muhammad Umer Suleman, Muhammad Mursaleen, Umer Khalil, Mohammad Zahir, Syeda Nafisa Tabassum

**Affiliations:** a Department of Internal Medicine, Ayub Medical College, Abbottabad, Pakistan; b Department of Microbiology, Bangladesh Rural Advancement Committee: BRAC

**Keywords:** clinical translation, ethical issues, organ shortage, organ transplantation, regenerative medicine, stem cell therapy, tissue engineering

## Abstract

Regenerative medicine offers hope to close the gap between organ failure and transplant availability, but progress remains incremental. This editorial reviews early clinical triumphs, such as cultured skin grafts and tissue-engineered bladders and vaginas, and contrasts them with persistent barriers to whole-organ replacement. We examine stem cell interventions that show functional gains in small trials (for example, cardiopoietic cells and stem-cell-derived islets) yet lack long-term evidence, consistent manufacturing, and proven survival benefit. Tissue engineering advances, including decellularization, perfusion bioreactors, and three-dimensional bioprinting, demonstrate feasibility for simple constructs but fall short for complex organs that require integrated vasculature and multiple specialized cell types. We highlight safety concerns such as thromboembolism and fibrosis, variable product quality, and the risk posed by unregulated clinics. Ethical, regulatory, and economic obstacles, including unclear approval pathways, absent long-term registries, high costs, and potential inequities, threaten translation. To advance patient-centered care, we call for rigorous and transparent clinical trials, shared manufacturing standards, mandatory long-term follow-up, and multidisciplinary collaboration focused on near-term clinically impactful strategies alongside long-term organ engineering research. Humility, vigilance, and alignment of innovation with clinical need are essential if lab-grown organs are to become safe and equitable therapies.

## Introduction

The gap between organ failure and transplant availability remains stark. Every day, patients die waiting for a transplant. For instance, by 2020, only about 32% of U.S. waitlist candidates received a donor organ^[1]^. Worldwide, the shortfall is even starker: roughly 150 000 solid organ transplants occur each year, barely 10% of global need^[2]^. This yawning gap has inspired hope that regenerative medicine can fill the void. Laboratory breakthroughs engineered skin grafts, lab-grown bladders, and autologous vaginas demonstrate that functional tissues can be grown and implanted^[3–5]^.

Yet we adopt a critical perspective. Are we truly on the brink of a new era in organ replacement, or are we mistaking piecemeal advances for a revolution? We juxtapose early successes with a frank discussion of obstacles. We scrutinize stem-cell therapies for organ repair, assess which tissue-engineering strategies might realistically reach patients versus those still speculative, and spotlight unresolved ethical, regulatory, and cost barriers. Throughout, we emphasize the need for a patient-centered approach grounded in realism and urgent clinical need. AI-assisted tools used in the preparation of this article were employed in accordance with the TITAN Guidelines 2025, as outlined in the TITAN Guideline Checklist^[6]^.

## Early triumphs and their limits

Regenerative medicine has yielded inspiring case studies, but they can create unrealistic expectations. Autologous skin culture is one of the earliest success stories: in 1981, O’Connor *et al* showed that a small biopsy of patient skin could be expanded into sheets covering large burns^[7]^. This procedure is now Food and Drug Administration–approved, saving lives and avoiding donor skin. More complex tissues have also been implanted. Anthony Atala’s team grew bladders from each child’s own cells; in seven children, these lab-grown bladders functioned like native organs^[4]^. Likewise, engineered vaginas have developed normal structure and function in young women^[5]^.

However, all of these cases involved ideal conditions: small cohorts of carefully chosen patients, use of autologous cells (eliminating rejection), and relatively simple hollow organ geometries. These procedures were slow, individualized, and have not been scaled up. They remain proof-of-concept anecdotes, not routine therapies. Crucially, they do not address the real challenge of complex organs: hearts, kidneys and livers have intricate vasculature and dozens of cell types, problems that are far from solved. Claims that whole organs will imminently be grown in the lab remain premature.

## The reality of stem cell therapies

Stem cell therapies hold great promise but face sobering limits. Some trials report modest gains: for example, one study infused “cardiopoietic” bone marrow cells into chronic heart failure patients and saw average ejection fraction rise from ~27% to ~34% (controls saw no change)^[8]^. In type-1 diabetes, an investigational stem-cell–derived islet transplant (VX-880) restored insulin production in all 12 trial patients and enabled 10 of 12 to become insulin-independent at 1 year^[9]^. These results hint that lab-derived cells can engraft and improve function in humans.

However, the hype must be tempered. Both cases were small, early-phase trials with no long-term follow-up; no stem-cell therapy is yet standard for organ failure. Larger analyses of cardiac cell therapy find only minimal benefit and no clear survival advantage. In wound healing, a 3-year follow-up found that while mesenchymal stem cells could heal diabetic foot ulcers, the regenerated skin remained abnormal, collagen density was ~57% lower and the epidermis ~72% thicker than normal^[10]^. In summary, stem cells often accelerate early repair but do not fully restore normal tissue architecture^[10]^.

Most critically, safety and consistency remain uncertain. Cell products vary widely in source and culture method, making outcomes unpredictable. Alarmingly, complications have been reported: thromboembolic events and fibrosis are among the most common adverse effects seen in mesenchymal stem-cell trials^[11]^. Such risks may appear months or years later, highlighting the need for rigorous trials, long-term monitoring and fully informed patient consent. In short, while stem-cell interventions remain scientifically intriguing, their scalability and reproducibility are far from proven.

## Bridging the gap: tissue engineering from lab to clinic

Modern engineering tools are impressive, but their clinical readiness varies. In the lab, researchers can decellularize whole organs and use perfusion bioreactors to keep constructs alive^[1,12]^. Even three-dimensional bioprinted tissues, for example, small cardiac patches or liver organoids, have been reported^[12]^. These advances hint at future possibilities. However, most remain experimental. To date, only relatively simple engineered tissues have reached patients (thin patches, hollow tubes, or cartilage shapes); building a fully functional organ is another matter. True organs require integrated vasculature and many specialized cell types, challenges that are only beginning to be addressed. Many ambitious ideas (bioprinted hearts, hybrid organs) remain speculative until major biological and engineering breakthroughs occur.

## Ethical, regulatory, and economic roadblocks

Even if scientific hurdles are overcome, societal challenges loom large. Regenerative therapies are likely to be extremely expensive: each lab-grown organ may cost many times a conventional transplant, raising equity concerns. Wealthy individuals or nations could monopolize access, widening global disparities.

Desperate patients might flock to unregulated “stem cell clinics” or risky cross-border transplants if proven options lag. The World Health Organization warns that organ scarcity already fuels unethical transplant tourism and trafficking, and unproven regenerative schemes could worsen this^[2]^.

Regulatory frameworks are also unprepared. Engineered organs blur lines between drugs, devices, and transplants, and many jurisdictions lack clear approval pathways. For example, should a bioprinted organ be regulated like a tissue graft, a biologic drug, or something new entirely? Long-term safety monitoring is a challenge: no current system tracks patients who receive experimental bioengineered tissues. Without international standards and mandatory registries, new therapies risk entering practice before being fully understood.

Finally, the cost barrier runs deep. So far, much work has been funded by governments and philanthropy, but few commercial models exist. Private insurers and health systems have little incentive to cover ultra-expensive, unproven treatments. If each engineered organ remains a custom six-figure experiment, it cannot alleviate the organ shortage it aims to solve.

## Conclusion

Every day, dozens of patients die for lack of a transplant. This urgency must drive regenerative research but not at the expense of realism. The field’s achievements – engineered skin, bladder and vaginal organs, and promising stem-cell infusions – show that radical ideas can yield real patient benefit. Yet progress remains incremental. Patients and physicians should not mistake hopeful press releases for proof.

Going forward, we must refocus on patient-relevant goals. This means rigorous, transparent clinical trials and global collaboration. It means developing shared standards for cell and tissue manufacturing, mandatory long-term follow-up, and fair allocation. It means multidisciplinary teams, surgeons, bioengineers, ethicists and regulators, working together to translate lab discoveries into safe therapies. Funding bodies and policymakers should prioritize approaches that offer near-term clinical impact (better donor organ use, bioactive wound matrices, etc.) alongside long-term organ engineering research.

Above all, we call for humility and vigilance. Acknowledge unknowns, learn from setbacks, and always put patient welfare first. Regenerative medicine is too important to be derailed by hype or dashed hopes. If we align innovation with clinical need and ethical principles, we can steadily approach a future where lab-grown organs save lives. This is a patient-centered challenge and a call to action for our entire community.

## Data Availability

All data generated during this study are included in this published article.
